# Crystal structure of a blue laccase from *Lentinus tigrinus*: evidences for intermediates in the molecular oxygen reductive splitting by multicopper oxidases

**DOI:** 10.1186/1472-6807-7-60

**Published:** 2007-09-26

**Authors:** Marta Ferraroni, Nina M Myasoedova, Vadim Schmatchenko, Alexey A Leontievsky, Ludmila A Golovleva, Andrea Scozzafava, Fabrizio Briganti

**Affiliations:** 1 Department of Chemistry University of Florence, Via della Lastruccia 3, Sesto Fiorentino, 50019 Florence, Italy; 2 G. K. Skryabin Institute of Biochemistry and Physiology of Microorganisms Russian Accademy of Science, Pushchino, Moscow Region, 1422920, Russia

## Abstract

**Background:**

Laccases belong to multicopper oxidases, a widespread class of enzymes implicated in many oxidative functions in pathogenesis, immunogenesis and morphogenesis of organisms and in the metabolic turnover of complex organic substances. They catalyze the coupling between the four one-electron oxidations of a broad range of substrates with the four-electron reduction of dioxygen to water. These catalytic processes are made possible by the contemporaneous presence of at least four copper ion sites, classified according to their spectroscopic properties: one type 1 (T1) site where the electrons from the reducing substrates are accepted, one type 2 (T2), and a coupled binuclear type 3 pair (T3) which are assembled in a T2/T3 trinuclear cluster where the electrons are transferred to perform the O_2 _reduction to H_2_O.

**Results:**

The structure of a laccase from the white-rot fungus *Lentinus (Panus) tigrinus*, a glycoenzyme involved in lignin biodegradation, was solved at 1.5 Å. It reveals a asymmetric unit containing two laccase molecules (A and B). The progressive reduction of the copper ions centers obtained by the long-term exposure of the crystals to the high-intensity X-ray synchrotron beam radiation under aerobic conditions and high pH allowed us to detect two sequential intermediates in the molecular oxygen reduction pathway: the "peroxide" and the "native" intermediates, previously hypothesized through spectroscopic, kinetic and molecular mechanics studies. Specifically the electron-density maps revealed the presence of an end-on bridging, μ-η_1_:η_1 _peroxide ion between the two T3 coppers in molecule B, result of a two-electrons reduction, whereas in molecule A an oxo ion bridging the three coppers of the T2/T3 cluster (μ3-oxo bridge) together with an hydroxide ion externally bridging the two T3 copper ions, products of the four-electrons reduction of molecular oxygen, were best modelled.

**Conclusion:**

This is the first structure of a multicopper oxidase which allowed the detection of two intermediates in the molecular oxygen reduction and splitting. The observed features allow to positively substantiate an accurate mechanism of dioxygen reduction catalyzed by multicopper oxidases providing general insights into the reductive cleavage of the O-O bonds, a leading problem in many areas of biology.

## Background

Laccases (benzenediol oxygen oxidoreductase; EC 1.10.3.2) are glycosilated multicopper oxidases. Since their discovery more than one century ago in the Japanese tree *Rhus venicifera *[1], laccases have been found to be widely distributed among plants, where they are involved in the wounding response and the synthesis of lignin, and subsequently they were discovered to be present in insects, bacteria and widely diffused in basidiomyceteous and ascomyceteous fungi [2].

The simplest reactions catalyzed by laccases are those in which a vast set of substrates, typically phenols and arylamino compounds, is oxidized to the corresponding radical species by direct interaction with their active site [3] and accompanied by the reduction of molecular oxygen to water. Due to their very broad substrate range they are implicated in a extensive series of functions such as pathogenesis, immunogenesis and morphogenesis of organisms and in the metabolic turnover of complex organic substances such as lignin, humic matter, and toxic xenobiotics [4].

For instance, white-rot fungi degrade wood lignin using a combination of specialized intra- and extra-cellular enzymes [5]. Lignin, the most common polymer on earth which provides the structural component of the plant cell wall, is a heterogeneous biopolymer composed by phenyl propanoid units linked by various non-hydrolyzable C-C- and C-O- bonds [6]. The ligninolytic system of white-rot fungi was thought to be mainly composed by lignin-peroxidase and manganese-peroxidase [7]. Laccases, incapable of directly cleaving the non-phenolic bonds of lignin, were not considered as significant components of the ligninolytic system, despite the secretion of large quantities of laccases by the vast majority of white-rot fungi under ligninolytic conditions. More recently it was discovered that white-rot fungi laccases can enlarge their substrate range and are then able to oxidize compounds with a redox potential exceeding their own, such as non-phenolic benzylalcohols [8-10]. In fact their catalytic competences can be further extended to substrates which are not oxidized directly, either because they are too large to penetrate into the enzyme active site or because they have a redox potential higher than the laccase itself. Nature overcomes these limitations with the utilization of mediators, which are suitable compounds that act as intermediate substrates for the laccase: the oxidized radical forms of which are sufficiently stable to leave the enzyme site and react with the bulky or high redox-potential substrate targets. This finding led to the discovery that laccase-mediator systems effectively play a major role in the biodegradation of lignin and recalcitrant aromatic pollutants [10-12]. These non enzymatic routes of oxidative polymerizing or depolymerizing reactions are vital in a range of physiological functions such as lignolysis, lignin synthesis, morphogenesis, pathogenesis and detoxification.

Owing to their high and non-specific oxidation capacities, to the lack of a requirement for cofactors and to the use of readily available oxygen as an electron acceptor laccases are useful biocatalysts with some established and lots of emerging biotechnological applications such as biobleaching, xenobiotics bioremediation, textile dyes decolorization, biosensors, food industry etc. [13-15].

These multicopper oxidases are generally monomeric glycoproteins containing about 500 amino acids arranged in 3 β-barrel domains assembled to model three spectroscopically distinct copper binding catalytic sites: one type 1 (T1, blue copper, characterized by a strong absorption at 600 nm), one type 2 (T2, normal copper) and one type 3 (T3, EPR-silent antiferromagnetically coupled dinuclear coppers)[16,17]. Four one-electron oxidations of the reducing substrates mentioned above which occur at the T1 site on the protein surface are coupled to the four-electrons reduction of dioxygen to water which occurs at the internal T2/T3 cluster [18,19]:

O_2 _+ 4 e^- ^+ 4 H^+ ^→ 2 H_2_O

The unique kinetic and spectral features of copper/dioxygen intermediates in multicopper oxidases have been investigated into details mainly by Reinhammar and Solomon groups [18,20]. These studies led to several hypotheses of molecular mechanisms for the four-electrons reduction of dioxygen to water by this class of enzymes.

X-ray structural studies on ascorbate oxidase, ceruloplasmin and on several fungal and bacterial laccases have further allowed the rationalization of important structural and functional aspects of multicopper oxidases such as the positioning of the T1 site, the electron transfer pathways between T1 and the T2/T3 cluster coppers, the oxygen and water channels, and some of the substrates/inhibitor interactions [16,19,21-28].

Regarding fungal laccases at first, the extensive microheterogeneity, presumably caused by variable glycosylation of these enzymes, hindered successful crystallization but deglycosylation performed to obtain high quality diffracting crystals resulted in the loss of copper as in the case of Ducros *et al*. which reported the crystal structure of a laccase from the fungus *Coprinus cinereus *at 1.68 Å resolution[16] in a form devoid of the type 2 copper and therefore in a catalytically incompetent state. More recently the x-ray structures of laccases from the fungi *Trametes versicolor *(1.9 Å resolution), *Melanocarpus albomyces *(2.4 Å resolution), *Rigidoporus lignosus *(1.7 Å resolution) *Cerrena maxima *(1.9 Å resolution), and the spore-coat laccase from *Bacillus subtilis *(1.7 Å resolution) have been described [21-24,29].

Substrate and dioxygen binding was investigated for the *Bacillus subtilis *endospore-coat laccase (substrate: ABTS) and for the *Trametes versicolor *laccase (substrate: 2,5-xylidine) [30,31].

Nevertheless several focal points still need to be elucidated from the structural standpoint including the nature of the intermediates in the oxygen reduction pathway.

In particular the biotechnological application of laccases, aiming at the development of various industrial oxidative processes going from the production of fine chemicals and pharmaceuticals to bioremediation of contaminated soil and water, would receive great impulse to improvements from the complete comprehension of the catalytic mechanism of multicopper oxidases, and in particular of their redox potential and substrate selectivity control and a detailed characterization of the high resolution molecular structure of such enzymes will surely help in achieving such aims.

We present here the structural analysis at the highest resolution available to date of an active laccase from *Lentinus tigrinus *(hereafter *Lt*L) a white rot lignin decomposing fungus able to biodegrade high concentrations of mixtures of chlorophenols such as 2,4-dichlorophenol, 2,4,6-TCP, and pentachlorophenol [32,33]. Furthermore the particular conditions utilized for x-ray diffraction data collection have allowed the detection of the structure of two sequential intermediates in the molecular oxygen reduction pathway: the "peroxide" and the "native" intermediates the latter only hypothesized through spectroscopic, kinetic and molecular mechanics calculations studies.

## Results and discussion

### Primary sequence

**Figure 1:**
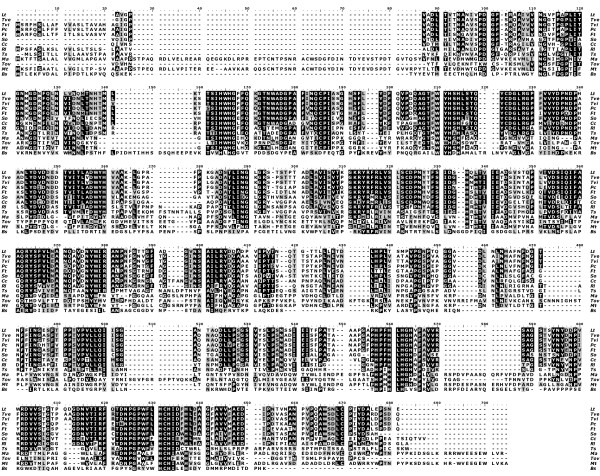
Alignment of the *Lt*L blue laccase amino acid sequence with other laccase sequences. Lt – *Lentinus (Panus) tigrinus *laccase [AAX07469 and Pdb code: 2QT6]; Tve – *Trametes versicolor *[gi:21730581, pdb:1KYA]; Tvi – *Trametes villosa *[AAC41686]; Pc – *Pycnoporus cinnabarinus *[AAF13052]; Ft – *Funalia trogii *[CAC13040]; So – *Steccherinum ochraceum *laccase [unpublished gene-xray]; Cc – *Coprinus Cinereus *[pdb:1A65]; Rl – *Rigidoporus Lignosus *[pdb:1V10]; Ts – *Thizoctonia solani *[CAA91042]; Ma – *Melanocarpus albomyces *[CAE00180]; Tov – *Toxicodendron vernicifluum *[BAB63411] ; Mt – *Myceliophthora thermophila *[AAC93841]; Bs – *Bacillus subtilis *Cota [pdb:1HKZ]. Positions identical in all sequences are marked with a black background. Regions, in which the sequences are similar are marked with light grey.

The nucleotide sequence of *L. tigrinus *laccase gene was obtained as reported in the experimental procedures, the deduced sequence resulted to be composed by 463 amino acids (accession code: AAX07469). Since the electron density maps are of very good quality the polypeptide chain has been traced throughout the molecule. This allowed us to obtain the entire sequence from the x-ray data, this resulting in a 498 amino acids chain (see Figure 1). The comparison of the sequence derived from the gene and that obtained from the x-ray data reveals that in the DNA sequence the residues corresponding to the c-terminal end and exactly from positions 464 to 498 were missing: VVMAEDIPNTVNANPVPQAWSNLCPTYDALEPSNE. The sequence of *L. tigrinus *blue laccase gene lacks some 3' end nucleotides as in the gene amplifying we have used the reverse primer based on internal conservative C-terminal amino acid sequence of basidiomycete laccases. Therefore the deduced amino acid sequence of *L. tigrinus *blue laccase is missing C-terminal residues. Furthermore the electron density maps allowed to correct the identity of a few other residues: in position 16: V is observed instead of S, in position 164: K is present instead of L, and in position 459: D occurs instead of E.

### Overall structure

**Figure 2:**
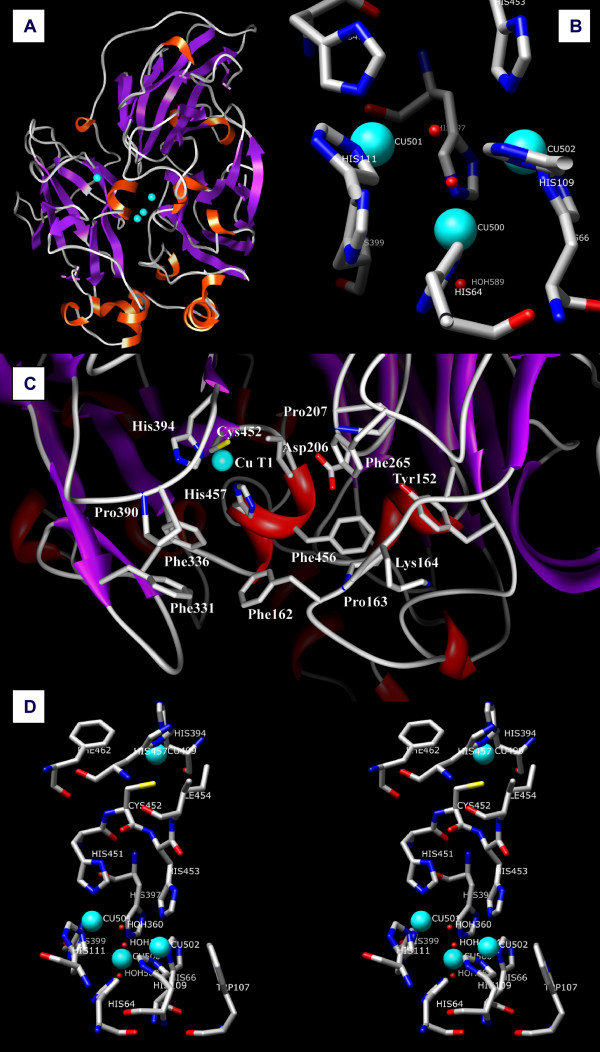
(A) Schematic representation of the structure of *Lt*L; the copper ions are depicted as magenta spheres. (B) T2/T3 trinuclear cluster active site as observed in molecule A of *Lt*L. (C) Substrate active T1 pocket residues. (D) Stereoview of the schematic representation of the four copper sites in *Lt*L.

The crystal structure of *Lt*L was solved at 1.5 Å resolution by using the molecular replacement technique and employing, as a starting model, the coordinates of the *Coprinus cinereus *laccase (pdb code: 1HFU). The schematic structure and folding topology of *Lt*L, a monomeric globular glycoprotein with overall dimensions 70 × 60 × 50 Å, is shown in Figure 2A. It exhibits a molecular architecture organized in three sequentially arranged cupredoxin-like domains; each of them has a *greek key β-barrel *topology, strictly related to the small copper proteins like azurin and plastocyanin and common to all the members of the blue multicopper oxidase family, like the ascorbate oxidase and the mammalian ceruloplasmin. Domain 1 includes residues 1–141, domain 2 residues 142–303, and domain 3 residues 304–498. As observed in Figure 1*Lt*L exhibits relevant structural similarities with several basidiomyceteous fungal laccases and particularly with the enzyme from *Trametes versicolor *[23] (78% of sequence identity), the superposition of the Cα main chain atoms of the two structures gives an rmsd of 0.515 Å. On the contrary major differences are noticed in the comparison with laccases from plants, bacteria and even with ascomyceteous fungi (see Figure 1).

Of the six putative N-glycosylation sites (consensus sequence N-X-T/S) only at Asn54, Asn376, and Asn435 the density maps indicate the presence of glycosilaytion. The corresponding electronic densities were modelled to two di(n-acetyl-D-glucosamine) and five branched α-D-mannose moieties bound to Asn54, one n-acetyl-D-glucosamine bound to Asn376 (but detected only in molecule A) and one di(n-acetyl-D-glucosamine) moiety bound to Asn435.

The structure is further stabilized by two sulphur bridges, one between Cys85 and Cys487 from domains 1 and 3 respectively and the other between Cys117 and Cys 205 from domains 1 and 2 correspondingly.

### Substrate binding site

The structure of *Lt*L does not exhibit any peculiar feature at the T1 active site of the enzyme where the reducing substrate binds to transfer electrons to the T1 copper, located in domain 3. The binding pocket for reducing substrates of *Lt*L is a relatively large superficial zone near the T1 copper depicted in Figure 2C. A number of residues mainly His457, the solvent exposed T1 copper ligand, and several surrounding amino acids are potentially involved in the interactions with the different substrates which are oxidized at that site; hydrophobic residues such as Phe162, Phe265, Phe331, Phe 336, Phe 456, are found together with hydrophilic residues like Asp206, Lys164, Tyr152, and several prolines: Pro163, Pro207, Pro390. The variety of these residues explains the large substrate specificity common to this type of enzymes and mainly related to the redox potential of the T1 copper ion (> 700 mV) which is comparable to several known high potential laccases comprising that from *T. versicolor *[23].

Two solvent channels provide access to the trinuclear copper cluster which is located in the interior of the protein structure. The first channel points towards the two T3 copper ions on one side of the T2/T3 cluster allowing to the molecular oxygen to bind to it whereas the second channel pointing towards the T2 copper ion on the other side of the cluster permits to the water molecules produced in the O_2 _reduction to move to the bulk solvent [22].

### Copper sites

The T1 copper ion (occupancy 0.75) presents a planar triangular coordination with two histidine (His394 and His457) and one cysteine (Cys452) residues. Two hydrophobic residues (Ile454 and Phe462) at about 3.5–3.6 Å distance (see Table 1) from the copper ion are thought to contribute to the high T1 copper redox potential observed for this enzyme (Figures 2C and 2D). In the catalytic process the electrons acquired by the T1 copper from the reducing substrates are then transferred to the T2/T3 cluster through an intramolecular electron transfer pathway starting from the T1 copper ligand Cys452 and subsequently splitting between His451 and His453 which bind to the T3(a) and T3(b) copper ions respectively [34].

**Table 1:** Observed coordination distances (Å) in the copper centers, copper-copper and oxygen-oxygen distances for *Lentinus tigrinus *laccase

**Molecule A**	**Molecule B**
**Mononuclear copper center: T1 copper**	

His394 ND1 2.03	His394 ND1 2.04
Cys452 SG 2.23	Cys452 SG 2.25
His457 ND1 2.01	His457 ND1 2.01
Phe462 CD2 3.68	Phe462 CD2 3.68
Ile454 CD1 3.60	Ile454 CD1 3.57

**Trinuclear copper center T2/T3**	

T3(a) copper	

His111 NE2 2.03	His111 NE2 1.99
His451 NE2 1.99	His451 NE2 2.02
His399 NE2 1.99	His399 NE2 2.00
OH O1 2.16	Perox O1 2.08
Oxo O2 2.91	Perox O2 2.80

T3(b) copper	

His453 NE2 2.11	His453 NE2 2.10
His66 ND1 1.99	His66 ND1 1.99
His109 NE2 2.01	His109 NE2 2.00
OH O1 2.95	Perox O1 3.08
Oxo O2 2.70	Perox O2 2.18

T2 copper	

His397 NE2 1.90	His397 NE2 1.91
His64 NE2 1.92	His64 NE2 1.91
OH 2.43	OH 2.49
OH O1 4.10	Perox O1 3.06
Oxo O2 2.09	Perox O2 4.25

Copper-copper	

T1-T3(a) 12.23	T1-T3(a) 12.24
T1-T3(b) 13.13	T1-T3(b) 13.14
T1-T2 14.85	T1-T2 14.86
T3(a)-T3(b) 4.94	T3(a)-T3(b) 4.91
T2-T3(a) 4.32	T2-T3(a) 4.32
T2-T3(b) 4.11	T2-T3(b) 4.09

Oxygen1-Oxygen2	

O1-O2 2.05	Perox O1-O2 1.43

Multicopper oxidases are easily reduced by addition of substrates or artificial reductants but their kinetics under aerobic conditions are complex and difficult to interpret, therefore the common strategy for the study of their catalytic mechanism has been first the analysis of the anaerobic reduction steps followed then by the investigation of oxygen reaction stages with the reduced enzyme [20].

A recently discovered alternative for the progressive reduction of metalloproteins is the exposure of crystals to a high-intensity X-ray synchrotron beam. In fact, in several instances, it has been observed that synchrotron radiation can yield the partial or total reduction of the metal ions depending on the particular metalloprotein studied, on the data collection method and accordingly on the acquired radiation dose [35,36]. This strategy, applied to multicopper oxidases, could allow to observe the progressive reduction of molecular oxygen and monitor the nature of the intermediate states. Up to date no structural studies allowed to perceive the different intermediates mainly because these states are generally short living and difficult to trap unless the further catalytic step is inhibited. The pH optima for *Lt*L are ranging from 2.3 for ABTS (2,2'-azinobis(3-ethylbenzthiazolinesulfonic acid), a non phenolic substrate, to pH 4.5 and 5.0 for 2,6-dimethoxyphenol and syringaldazine respectively. Furthermore it is well known that the activity of laccases is inhibited at high pH values due to the formation of a strong T2 Cu-OH^- ^complex which hinders the subsequent transformation of reduced oxygen to water molecules (O^2- ^+ 2H^+ ^→ H_2_O) [37-39]. For these reasons the present experiment was designed to be performed at high pH in order to allow the trapping of at least the completely reduced state of the oxygen-enzyme adduct.

Exposing *Lt*L crystals at pH 8.5 to high X-rays radiation doses under aerobic condition allowed us to detect two intermediates in the catalyzed molecular oxygen splitting, most likely corresponding to the 2-electrons and 4-electrons reduction stages.

### The 2-electrons reduction intermediate

The electron-density maps from *Lt*L disclosed two different T2/T3 cluster-dioxygen arrangements in the two laccase molecules (A and B) present into the asymmetric unit, possibly corresponding to two different reduction states of the oxygen molecule along the reduction pathway. In both molecules the T2 copper center is coordinated to His64, His397 and one OH^- ^ligand whereas the T3 copper centers are coordinated to 3 histidine residues each (His111, His399, and His451 for T3(a), His66, His109, and His453 for T3(b))(See Figures 2B and 2D).

**Figure 3:**
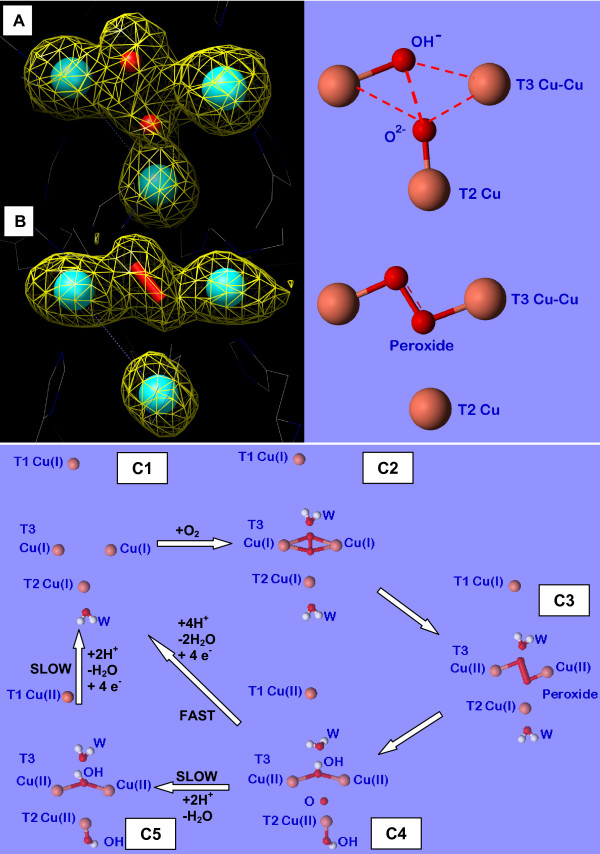
(A) and (B) Representations of the Fo-Fc difference Fourier omit map for the T2/T3 active site of molecules A and B of *Lt*L respectively, each flanked by the corresponding schematic pictures. The electron density is contoured at 2.2 σ. (C) Schematic representation of the catalytic mechanism of multicopper oxidases including the intermediates observed in the present structural study and previous spectroscopic, kinetic, and structural investigations.

**Figure 4:**
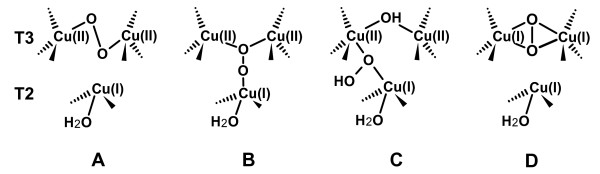
Schematic representations of possible adducts of the trinuclear T2/T3 copper cluster with 2-electrons reduced dioxygen (peroxide).

It should be noted that differences observed among the structural data of multicopper oxidases published to date, and acquired using synchrotron radiation, are difficult to interpret since they most probably represent pictures of the copper sites resulting from the average of the starting oxidized state and of the partially/totally reduced coppers/dioxygen adduct states and strictly reliant on the acquired x-ray doses and on the other experimental conditions. From the distances reported on Table 1 it can be deduced that in the present structure we mainly observe a fully reduced structure, the inhibition of the hydroxide/water formation allowed to detect in molecule B an electronic density compatible with the presence of an end-on bridging μ-η_1_:η_1 _peroxide moiety (O1-O2 distance 1.43 Å) between the two T3 coppers (see Figures 3B, and 4A) [see also additional file 2]. This peroxide ion should be the result of the two-electrons reduction of dioxygen. Previous kinetic and spectroscopic investigations have led to describe the four-electrons reduced native laccase as the species that binds dioxygen at the T3 copper pair then generating the first intermediate in dioxygen splitting: two electrons are transferred to O_2 _from the T3 copper centers generating a peroxide intermediate [18,20]. A ligand-field analysis also suggested that the T2 copper site becomes four coordinated in the peroxy-bound form probably due to the peroxide forming a bridge between the T2 and the T3 copper centers. These studies headed to two possible descriptions of the peroxide intermediate in the trinuclear copper cluster. In one model, the peroxide internally bridges the T2 to the T3 copper centers with the magnetic coupling between the oxidized T3 Cu centers arising from antiferromagnetic coupling through one oxygen atom of the peroxide group (Figure 4B). Alternatively, the T3 Cu(II) centers could be linked by the hydroxide bridge which is also present in the resting state and the peroxide could externally bridge the T2 Cu(I) and the T3 Cu(II) centers as hydroperoxide (Figure 4C). Only very recently a computational method that combines extended X-ray absorption fine structure (EXAFS) refinements with an integrated quantum mechanical and molecular mechanics method allowed to suggest that the structure with a peroxide ion in the center of the trinuclear cluster fits experimental data better than the other model [40].

Dioxygen moieties have been observed in several previously collected structural data on multicopper oxidases [24,41,42]. Since strong molecular oxygen binding is feasible only when copper is in the Cu(I) oxidation state these findings indicate that the x-ray sources utilized in the corresponding experiments have been able to reduce at least the T3 copper pair. Whereas in the ABTS substrate adduct of *B. subtilis *CotA laccase [31] the dioxygen is found within the oxygen channel oriented toward one of the T3 copper ions, in *M. albomyces *laccase and *B. subtilis *CotA CuCl_2 _soaked or M502L mutant laccases, dioxygen is bound to both T3 copper ions in an nearly symmetrical side-on μ-η^2^:η^2 ^binding mode (Figure 4D) [24,41,42].

Furthermore, structures of adducts with exogenous peroxide have been obtained with ascorbate oxidase from zucchini and CotA laccase from *B. subtilis*. The structure of the CotA laccase shows the peroxide molecule asymmetrically bound between the two T3 copper ions in a end-on bridging μ-η_1_:η_1 _mode [41] (Figure 4A), thus agreeing with the observations of the present investigation for molecule B of *Lt*L, the only difference being that in the present structure the peroxide ion has been generated by internal two-electrons dioxygen reduction. A similar peroxide adduct was also observed in the structure of the untreated *B. subtilis *CotA laccase M502F mutant but no possible rationalization was reported for such finding [42]. On the contrary in ascorbate oxidase the peroxide ion was located terminally bound to only one of the T3 copper ions and oriented along the oxygen channel [43].

### The 4-electrons reduction intermediate

**Figure 5:**
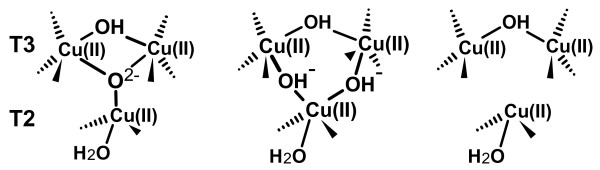
Schematic representations of different possible adducts of the trinuclear T2/T3 copper cluster. A and B: 4-electrons reduced dioxygen adducts, C: enzyme resting state.

In molecule A of *Lt*L the x-ray irradiation resulted in an electron density map that can be interpreted with the appearance of an oxo ion (occupancy 0.5) in the middle of the T3/T2 cluster bridging all the three coppers (μ3-oxo bridge with distances: O-T3(a)Cu 2.91 Å, O-T3(b)Cu 2.70 Å, and O-T2Cu 2.09 Å) together with an hydroxide ion (occupancy 0.75) asymmetrically bridging the two T3 coppers (distances: OH-T3(a)Cu 2.16 Å, and OH-T3(b)Cu 2.95 Å) possibly the products of the four-electrons reduction of molecular oxygen (Figures 3A and 5A) [see also additional file 2].

In particular in molecule A the reported distances would suggest that the electron density picture observed could be the outcome of an average between the two main intermediates: peroxide and hydroxyl/oxo ions. Indeed the distance between the modeled hydroxide and oxo ions resulted to be 2.05 Å; although this value does not correspond to a completely broken bond between the two oxygen ions it surely indicates the progress towards the breaking of the O-O bond and the formation of an oxo moiety (actually, in molecule B we observe an oxygen-oxygen average distance of 1.43 Å which is consistent with a peroxide ion). Unfortunately the attempt of separating the contribution of the peroxide and oxo/hydroxyl forms as well as a rigorous analysis of single bond distances would result in an over-interpretation of the available data.

The step resulting from the peroxide ion reduction to the H_2_O/OH^-^/O^2- ^level, the so-called "native" or "optical" intermediate, was previously investigated through spectroscopic and kinetic experiments. X-ray edge, MCD spectroscopy and magneto-structural correlations showed that the unusual spectra of the native intermediate are associated with a fully oxidized trinuclear copper cluster with all three copper centers involved in strong bridging interactions [44]. These observations conducted Solomon and coworkers to two possible structural models of the native intermediate where the four-electrons reduction of dioxygen has either produced one μ_3_-oxo bridge or two additional OH^- ^bridges (Figures 5A and 5B respectively). More recently Rulisek et al. suggested through quantum and molecular mechanical calculations that the μ_3_-oxo bridge should be the product of O-O bond cleavage [45,46]. Unfortunately no structural evidences were available to confirm it. Our data shed light on the structure of the "native" intermediate.

In *Lt*L the T3(a) and T3(b) copper ions are not symmetrically coordinated to the oxygen intermediates in both molecules A and B. Also in the *Rigidoporus lignosus *laccase (*Rl*L) structure [21] it has been recently observed the asymmetric binding of the hydroxide ion bridging the T3 copper ions (The T3(a)-OH bond being shorter than the T3(b)-OH bond); this was explained by assuming a reduced oxidation state for T2 and T3(b) copper ions, probably caused by autoreduction phenomena induced by the extensive exposure to high source of X-ray radiation. This asymmetry could have a crucial role in the progression of the dioxygen splitting. It has to be noted that the T3(a) copper results to be about 0.9 Å closer to the T1 site than the T3(b) copper. According to the theoretical calculations performed by Kyritsis et al. [34] for ascorbate oxidase, the particular conformation of the interposed aminoacidic residues, also observed in *Lt*L, should render the T1-T3(a) pathway up to three times more efficient than the T1-T3(b), thus explaining a differential reduction progress in the T2/T3 cluster possibly resulting in the observed asymmetries.

In the reported investigation we have been able to discern both the peroxide and the native intermediates resulting from the two- and four-electrons molecular oxygen reduction respectively, starting from aerobic crystals in which the complete dioxygen turnover is inhibited by the high pH due to the formation of a strong T2 Cu-OH^- ^complex which hinders the subsequent transformation of reduced oxygen to water molecules (O^2- ^+ 2H^+ ^→ H_2_O) [37-39].

The structural characterization of these new intermediates in the reduction of O_2 _to H_2_O in the multicopper oxidases leads now to propose a more detailed unified molecular mechanism (Figure 3C). As in several previously reported laccase structures the presence of the dioxygen moiety indicates that at least the reduction of the T3 copper pair has occurred. Once reduced (Figure 3-C1) the enzyme reacts with molecular oxygen (Figure 3-C2) to successively generate the peroxide intermediate (Figure 3-C3). Contrarily to what suggested by spectroscopic studies in the crystal structures the peroxide appears to bridge only the T3 copper pair and not to connect the reduced T2 copper center (Figure 3B). This peroxide intermediate is further reduced in a second step, thus oxidizing the T2 and the distant T1 centers to generate the "native" intermediate (Figure 3-C4) which is composed by one μ_3_-oxo bridge between the T2/T3 copper centers of the trinuclear cluster and an OH- bridge between the two T3 ions (Figure 3A).

Since it is known that at high pH the formation of a strong T2 Cu-OH^- ^complex inhibits the laccase activity hindering the O^2- ^+ 2H^+ ^→ H_2_O reaction to occur at the trinuclear copper site [37-39], working at pH 8.5 allowed the trapping of the oxygen reduced intermediates.

Although the final electron density is an average of the different oxidation states starting from the "resting" completely oxidized state to the trapped reduced oxygen adducts and even if the experimental conditions utilized cannot allow to precisely quantify the contributions of the different states to the final picture it is noticeable that the occupancy of the oxo ion resulted to be about 0.5 suggesting that this transient species can be definitely trapped employing the above reported strategy.

Also at low pH, the kinetic data show that both the rate of decay of the native intermediate to form the resting oxidized enzyme (Figure 3-C5) and the rate of reduction of the resting enzyme are too slow to be consistent with the catalytic turnover rate [37], On the contrary the "native" intermediate is rapidly reduced by substrate in the catalytic reaction thus indicating that this is the fully oxidized form of the enzyme which is active in catalysis [44]; only in the absence of substrates it slowly decays to the resting state. The oxidized resting state (Figure 5C) has been usually observed in the structures of resting multicopper oxidases, among them ascorbate oxidase from zucchini, and laccases from *Trametes versicolor*, and *Bacillus subtilis *CotA [22,23,26]. All the copper ions are apparently in an oxidized state.

## Conclusion

Laccases belong to multicopper oxidases, an important and widespread class of enzymes implicated in a extensive series of oxidative functions in pathogenesis, immunogenesis and morphogenesis of organisms as well as in the metabolic turnover of complex organic substances such as lignin, humic matter, and toxic xenobiotics. They catalyze the coupling between four one-electron oxidations of a broad range of substrates with the four-electron reduction of dioxygen to water.

The structure of the blue laccase, a multicopper oxidase from the white-rot basidiomycete fungus *Lentinus (Panus) tigrinus*, involved in lignin and xenobiotics biodegradation, was solved at 1.5 Å. The reduction of the copper ions centers obtained by the long-term exposure of the crystals to the high-intensity X-ray synchrotron beam radiation in aerobic conditions and pH 8.5 allowed us to trap two intermediates in the molecular oxygen reduction pathway: the "peroxide" and the "native" intermediates result of a two- and four-electrons reduction of molecular oxygen, previously hypothesized through spectroscopic kinetic and molecular mechanics studies. The pictures rising from the present results allow the completion of the structural pictures for an accurate mechanism of dioxygen reduction catalyzed by multicopper oxidases providing general insights into the reductive cleavage of the O-O bonds, a leading problem in many areas of biology.

## Methods

### Microorganism

White rot basidiomycete *Lentinus *(*Panus*) *tigrinus *8/18 was isolated from rotting wood in Dushanbe (Tadzhikistan) and stored on malt agar slants.

### Cloning of the blue laccase gene

To obtain the nucleotide sequence of blue laccase gene, the specific genomic sequence of *L. tigrinus *was amplified. *L. tigrinus *genomic DNA was isolated from fungal mycelia grown in Soya-glycerol media [47]. Filtered mycelium was quick-frozen and ground in liquid nitrogen and genomic DNA was purified by the procedure as described [48]. Primers for amplification of blue laccase gene sequence were designed based on the N terminus sequence of blue laccase as previously reported [49] and of internal conservative C-terminus amino acid sequence of basidiomycete laccases. The forward primer was 5'-GCCGTCGGTCCTGTCGCCGA-3' and the reverse primers were 5'-TGGCAGTGGAGGAACCACGGGCC-3' and 5'-GGCGAAACCAGCCTCGAGGTGGAAGTC-3'. Amplification of the blue laccase gene sequence from *L. tigrinus *genomic DNA was performed using the forward primer and one of the reverse primers and Tli DNA polymerase (Promega). PCR conditions were a first denaturing step at 94°C for 5 min and 30 cycles of 94°C for 45 s, 65°C for 45 s and 72°C for 1 min in a GeneAmp 2400 thermocycler (PerkinElmer). PCR products were loaded on a 1% agarose gel, electrophoresed in Tris-acetate-EDTA buffer and a single PCR product of 2 kb was cut and purified with the Wizard PCR preps purification kit (Promega). A 2 kb PCR product corresponding to the blue laccase gene without the fragment encoding the signal peptide was cloned into the pBluescript II KS vector (Stratagene). Cloned specific blue laccase sequences from some independent PCR experiments were sequenced on both strands.

### DNA sequencing and analysis

DNA was sequenced with the Big Dye terminator ready reaction kit from PE-Applied Biosystems and the Applied Biosystems DNA sequencer ABI PRISM 310 Genetic Analyzer. Sequencing reactions were performed on Thermal Cycler 2700 (Applied Biosystems) according to the manufacturer's protocols. The sequence data were processed and the encoded amino acid sequences were predicted using the Vector NTI (InforMax, Inc.) software package. Multiple sequence alignments of amino acid sequences were performed by the CLUSTAL program [50]. The GenBank accession number for the sequence of *Lt*L is AAX07469.

### Protein purification

Enzyme preparations containing the blue laccase from *Lentinus tigrinus *were obtained from fungal cultures grown as previously described [49]. The blue laccase was purified to apparent electrophoretic homogeneity as reported and was used for subsequent crystallization experiments. Laccase activity was determined quantitatively by monitoring the oxidation of 0.2 mM ABTS (2,2_-azinobis(3-ethylbenzthiazolinesulfonic acid) at 420 nm (extinction coefficient, 36,000 mM^-1 ^cm^-1^) in the presence of 20 mM sodium acetate, pH 5.0 at 20°C.

### Metal content analysis

Analysis of the protein metal content was performed by using a PerkinElmer Optima 2000 Inductively Coupled Plasma AES (Atomic Emission Spectrometry) Dual Vision. The metal content analysis was performed in triplicate on re-solubilized crystals of LtL; it revealed the presence of 0.7–0.8 equivalents of copper ions per metal coordination site.

### Crystallization and data collection

Crystals of laccase from *L. tigrinus *grow within one week at 296 K using the sitting drop vapor diffusion method. 3 μl of a 20 mg/ml protein solution were added to 2 μl of a solution containing 22% PEG 4000, 0.2 M CaCl_2_, 100 mM Tris·HCl pH 8.5 [32]. Crystals belong to the monoclinic space group P2_1 _with unit cell dimensions a = 54.2, b = 111.6, c = 97.1, β = 97.7. Assuming two molecules per asymmetric unit the solvent content is 46% of the unit cell (Vm = 2.31 Å ^3^/Da). A complete native data set was collected at the BW7A beamline at the DORIS storage ring, Hamburg, Germany at the copper edge (1.377 Å). 358 images at 4000 KHz dose each, (for a total time of exposure of ~12 hours) were collected at 100 K adding 10% glycerol to the mother liquor as cryoprotectant at a maximum resolution of 1.50 Å. Data processing with Denzo and Scalepack gave 180651 unique reflections, an overall completeness of 99.1% and a R_sym _of 0.062 [51].

### Structure determination and refinement

Structure determination was carried out by the molecular replacement technique using the laccase from C. cinereus (PDB code 1hfu) as a search model (see Table 2). This enzyme shares 58% sequence identity with the *L. tigrinus *laccase. Water molecules, Cu atoms and sugars were omitted from the standard model, which was used within the program MOLREP to calculate cross-rotation and translation function in the 10-3 Å resolution range [52]. Two clear solutions were evident, giving an R factor and correlation coefficient of 0.469 and 0.436, respectively. The initial electron-density maps are very clear and show the presence of all four Cu atoms (see Fig. 2) and of several carbohydrate moieties.

**Table 2:** X-ray data collection and atomic model refinement statistics

Space Group	P2_1_
**Unit cell parameters**	
a (Å)	54.22
b (Å)	111.61
c (Å)	97.09
β (°)	97.75
Wavelength (Å)	1.377
Limiting resolution (Å)	25.3-1.5 (1.53–1.50)
Unique reflections	180651
R_sym_(%)^a^	0.062 (0.481)
Multiplicity	6.6
Completeness overall (%)	99.1 (97.2)
<I/s(I)>	20.3 (3.3)
**Refinement**	
Resolution range (Å)	25.3-1.5
Unique reflections, working/free	179673/916
Rfactor (%)^b^	15.0
Rfree(%)	18.2
Non-hydrogen atoms	9602
Water molecules	1660
r.m.s.d. bonds(Å)	0.015
r.m.s.d. angles (°)	1.671
<B>	24.3

Values in parentheses are for the highest resolution shell.

^a ^R_sym _= Σ | I - <I>|/Σ I

^b^R_factor _= Σ | F_obs_-F_calc_|/Σ F_obs_

Manual rebuilding of the model was performed using the program QUANTA [53]. Solvent molecules were introduced automatically using ARP [54]. Refinement of the model was performed using the program Refmac 5.1.24;[52] 916 reflections (5‰ of the data) were randomly excluded from the refinement and used to follow the progress of the procedure with R_free_. The final refinement resulted in R_factor _and R_free _values of 15.0 and 18.2%, respectively.

Sulfur atoms, copper, chloride, and calcium ions were refined anisotropically. The coordination distances for the copper ions were refined unrestrained. The occupancies of the copper atoms were adjusted so that their isotropic thermal parameters were refined to obtain values similar to the local average and taking into consideration the average content of copper ions as determined through metal content analysis. The resulting occupancies are 0.75 for the T1 and the two T3 copper ions, and 0.5 for the T2 copper ion [see additional file 1]. Data processing and refinement statistics are summarized in Table 2. The overall mean B factor of the structure after refinement was 17.69 Å^2 ^for chain A, 21.21 Å^2 ^for chain B, 40.39 Å^2 ^for water molecules and 24.33 Å^2 ^for all atoms. The stereochemistry was checked with the program PROCHECK [55]. The 86% of the protein residues lies in the most favourite regions of the Ramachandran plot and only one residue (Leu 57) is in a non allowed region.

The final model include two independent molecules (A and B) related by a binary axis, 8 rameic ions, 6 calcium ions, 3 chloride ions, 2 glycerol molecules, one tartrate molecule and 1660 solvent molecules.

The model also include a few N-linked oligosaccharides moieties at the following sites: A/B54, A376, A/B435. More specifically we found a di(N-acetyl-D-glucosamine) linked to residue Asn435, a N-acetyl-D-glucosamine linked to residue AsnA376 and a mannose-type glycan bound to residue Asn A/B54.

A double conformation was modeled for the side chains of residues: MetA40, SerA62, CysA117, CysA205, SerA235, Gln A293, Met A310, Glu A 494, Glu A498, Met B40, Cys B117, Val B231, Ser B367, Ser B375, Glu B494.

No evidences of crystal damage were observed after data collection completion.

Protein coordinates have been deposited with the Protein Data Bank (Protein Data Bank accession number 2QT6).

## Authors' contributions

MF, AS and FB conceived of the studies, performed the crystallographic experiments, the structural analysis and interpretation and the manuscript writing work, NMM, AAL, and LAG carried out the work on enzyme purification and biochemical characterization, VS performed the cloning and sequencing experiments. All authors read and approved the final manuscript.

## Supplementary Material

Additional file 2 Figure 6 – Representations of the 2Fo-Fc difference Fourier map with a superposed Fo-Fc difference Fourier map, for the T2/T3 active site of molecules A and B of *Lt*L. Figure 6 (A) and (B) shows the representations of the 2Fo-Fc difference Fourier map (cyan colored, the electron density is contoured at 3σ) with a superposed Fo-Fc difference Fourier map (red colored, The electron density is contoured at 3σ), for the T2/T3 active site of molecules A and B of *Lt*L respectively. Click here for file

Additional file 1 Table 3 – Statistical occupation and temperature B factors for the atoms composing the trinuclear copper cluster moiety for both molecules A and B.The Table reports the statistical Occupation and Temperature B factors for the atoms composing the trinuclear copper cluster moiety for both molecules A and B. Click here for file
