# Supplementary material for: Crystal structure of a blue laccase from Lentinus tigrinus: evidences for intermediates in the molecular oxygen reductive splitting by multicopper oxidases
Source: BMC Struct Biol. 2007 Sep 26;7:60. doi: 10.1186/1472-6807-7-60 (PMC2064922; doi:10.1186/1472-6807-7-60)
Supplement: Additional file 2 — Figure 6 – Representations of the 2Fo-Fc difference Fourier map with a superposed Fo-Fc difference Fourier map, for the T2/T3 active site of molecules A and B of LtL. Figure 6 (A) and (B) shows the representations of the 2Fo-Fc difference Fourier map (cyan colored, the electron density is contoured at 3σ) with a superposed Fo-Fc difference Fourier map (red colored, The electron density is contoured at 3σ), for the T2/T3 active site of molecules A and B of LtL respectively. [file 1472-6807-7-60-S2.pdf]

Supplementary Material 2

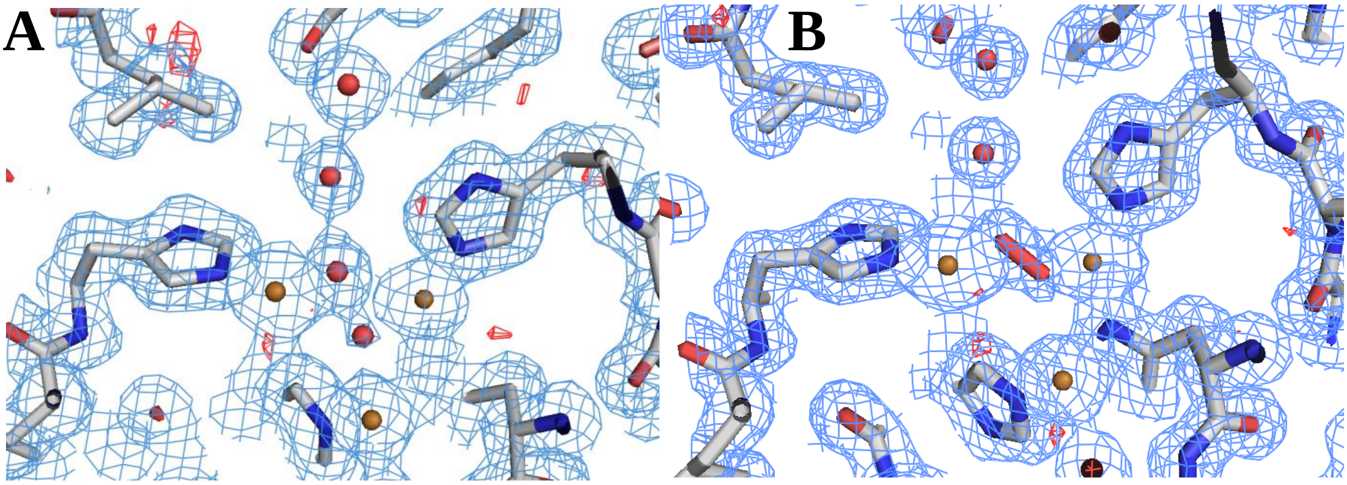

Figure 6 - (A) and (B) Representations of the 2Fo-Fc difference Fourier map (cyan colored, the electron density is contoured at 3σ) with a superposed Fo-Fc difference Fourier map (red colored, The electron density is contoured at 3σ), for the T2/T3 active site of molecules A and B of *Lt*L respectively.
