# Supplementary material for: Crystal structure of a blue laccase from Lentinus tigrinus: evidences for intermediates in the molecular oxygen reductive splitting by multicopper oxidases
Source: BMC Struct Biol. 2007 Sep 26;7:60. doi: 10.1186/1472-6807-7-60 (PMC2064922; doi:10.1186/1472-6807-7-60)
Supplement: Additional file 1 — Table 3 – Statistical occupation and temperature B factors for the atoms composing the trinuclear copper cluster moiety for both molecules A and B.The Table reports the statistical Occupation and Temperature B factors for the atoms composing the trinuclear copper cluster moiety for both molecules A and B. [file 1472-6807-7-60-S1.pdf]

Supplementary Material 1

Table 3

Occupation and Temperature B factors for the atoms composing the trinuclear copper cluster moiety for both molecules A and B

Atom	*Occupancy*	*B Factors ($Å^2$)*
**Molecule A**		
Cu1A	0.75	19.18
Cu2A	0.5	19.78
Cu3(a)A	0.75	22.30
Cu3(b)A	0.75	21.25
OH O1	0.75	21.79
Oxo O2	0.5	26.72
**Molecule B**		
Cu1B	0.75	24.22
Cu2B	0.5	23.87
Cu3(a)B	0.75	26.90
Cu3(b)B	0.75	24.14
Per O1	0.75	33.73
Per O2	0.75	23.57
